# The relationship between internal migration and the likelihood of high-risk pregnancy: Hukou system and high-risk pregnancies in China

**DOI:** 10.1186/s12884-021-03958-4

**Published:** 2021-07-15

**Authors:** Di Tang, Xiangdong Gao, Peter C. Coyte

**Affiliations:** 1 School of Public Administration, East China Normal University, 3663 N. Zhongshan Rd, Shanghai, 200062 China; 2 Shanghai First Maternity and Infant Hospital, Tongji University School of Medicine, No. 2669 Gaoke West Road, Pudong New Area, Shanghai, 201204 China; 3 Shanghai University of Medicine & Health Sciences, No. 279 Zhouzhu Highway, Pudong New Area, Shanghai, 201318 China; 4 Institute of Health Policy, Management and Evaluation, University of Toronto, 155 College Street, Suite 425, Toronto, ON M5T 3M6 Canada

**Keywords:** Within country migration, High-risk pregnancies, Healthy immigrant effect

## Abstract

**Background:**

China has one of the world’s largest internal migrant populations. The Chinese Hukou system is a unique household registration system that limits internal migrants in their access to basic urban public services, such as public health insurance and social assistance of their host city. In the case of female internal migrants, this may lead to high-risk pregnancies. The objective of this study is to assess the relationship between internal migrant status (Hukou) and the likelihood of high-risk pregnancies that occur in one large municipal-level obstetrics hospital in Shanghai, China.

**Methods:**

Medical records data from the Shanghai First Maternity and Infant Hospital from January 1, 2013, to May 31, 2018, were used to analyze 133,358 live births for Shanghai natives (*n* = 83,872) and internal migrant women (*n* = 49,486). A propensity score matching approach was used in conjunction with logistic regression analysis to identify the role of internal migrant status (Hukou) on the likelihood of high-risk pregnancies.

**Results:**

A greater likelihood of high-risk pregnancies were found among internal migrant women who moved from other parts of China to Shanghai. This effect was more obvious for women who gave birth for the first time and internal migrant women who were employed.

**Conclusion:**

The results show the effects of internal migrant status (Hukou) and the elevated likelihood of high-risk pregnancies among internal migrant women relative to their urban counterparts in Shanghai even after accounting for self-selection by employing the propensity score matching method. China’s unique Hukou household registration system limits access to public services for internal migrant women and accordingly may account for the elevated likelihood of high-risk pregnancies.

**Supplementary Information:**

The online version contains supplementary material available at 10.1186/s12884-021-03958-4.

## Background

There is an extensive literature that has addressed the health of international migrants [1–4]. Some studies have focused on the occurrence of high-risk pregnancies among these international migrants [5–9]. However, there is a paucity of literature examining perinatal health outcomes among migrants within a single country, in particular, China [10, 11]. In China, internal migrants may be at greater risk of adverse health events due to the presence of the Hukou household registration system that has been shown to limit access to a range of public services in their host cities, including health care services [12, 13]. This is especially true in large metropolitan areas like Shanghai where internal migrants, in 2017, accounted for 40% of the 24 million residents [14]. A previous study found that migrant mothers are more likely to have adverse birth outcomes than their counterparts [15].

The Hukou system, or household registration system, was set up by the Chinese government in 1958 to control a large internal migrant population. In China, locally born, urban residents have the right to enjoy the benefits of a series of national welfare programs (including medical insurance, housing, education, etc..), whereas internal migrants to these urban areas do not have access to these programs [10, 16–19]. Especially lack of medical insurance, where internal migrants tend to pay out of pocket for these costs, which may reduce their use of prenatal check-ups Recent research on the health status of Chinese internal migrants found internal migrants were more vulnerable to infectious and sexually transmitted diseases, occupational injuries and diseases, and had a greater likelihood of having poor reproductive health and high maternal mortality [20, 21]. When internal migrants are not eligible for public health insurance and assistance programs, they resort to paying out-of-pocket for such services [22]. Moreover, internal migrants, especially female internal migrants, have limited access to medical insurance and they often have low income, lower educational status, and frequently lack knowledge of antenatal care. All this taken together can result in an increased likelihood of high-risk pregnancies. A prior study found that health insurance coverage has improved infant health outcomes for migrant women in Shanghai [23].

The “healthy migrant effect,” whereby migrants are typically healthier than natives tends to suggest that migrants will have relatively positive health outcomes [13, 24–26]. However, evidence of the healthy migrant effect on perinatal outcomes is quite heterogeneous across different outcomes and highly context-dependent, and accordingly, country-specific [5, 9, 27–29]. Possible reasons for these observations were that the prior literature assumed migration was exogenous, ignored the heterogeneity of migrants [30, 31], and did not assess potential selection biases associated with the decision to migrate [31, 32]. The migration decision is influenced by the underlying health status of those that consider migration [25, 26]. Therefore, the standard application of regression analysis might be biased because the sample of migrants may be systematically different, for selection reasons, than all individuals who may have considered migration. These statistical challenges are addressed in this manuscript through the use of a popular econometric tool, that is, the propensity score matching (PSM) method [32, 33].

The key objective of this study was to use propensity score matching techniques to control for potential self-selection into internal migration and potential confounding of the Hukou system to assess the independent impact of internal migrant status (Hukou) on the likelihood of high-risk pregnancies in China, thereby controlling for the range of statically challenges not previously assessed in the literature. The data were drawn from all inpatient births over the period January 1, 2013, to May 31, 2018, at the Shanghai First Maternity and Infant Hospital. This hospital is one of the largest obstetrical hospitals in China with the highest annual number of births (more than 30,000).

To the best of our knowledge, this is the first study to measure the effect of internal migrant status (Hukou) on the likelihood of high-risk pregnancies in China. In the next section, we describe the data and methods. Results section reports our results and sensitivity tests are discussed in Sensitivity tests section. Our findings are discussed in the context of the literature in Discussion section. Conclusion section offers a brief set of conclusions and policy implications.

## Methods

### Data

This study used inpatient hospital admission data on all women who gave birth between January 1, 2013 and May 31, 2018 at the Shanghai First Maternity and Infant Hospital. These data were used to evaluate maternal high-risk pregnancies. The data contains patient demographics and clinical details on 133,358 live births, with 37% being internal migrants. Our study was approved by the Shanghai First Maternity and Infant Hospital.

### Dependent variable

High-risk pregnancy status was identified in the medical records and was defined by the National Health Commission of the People’s Republic of China [34]. Specifically, high-risk pregnancy refers to a situation wherein either the mother or the baby were more likely to have health problems during the course of pregnancy, including medical risk, patients with significant medical and surgical disorders, such as chronic hypertension, diabetes, cardiac disease, gastrointestinal disease, etc., and obstetric risk, healthy gravidas with fetuses at increased risk of adverse outcomes, such as multiple gestations, prior intrauterine fetal demise, isoimmunization, etc. This variable was coded as 1 when a mother/fetus had any one of the aforementioned high-risk pregnancies and 0 otherwise [35]. A wide variety of medical and/or obstetric complications are internationally-accepted as high-risk pregnancies during pregnancy, including preeclampsia, gestational diabetes, placenta previa, and fetal problems [36].

### Key independent variable

Although most internal migrants have rural or urban hukou in their home towns, a host urban hukou remains a crucial determent for internal migrants to access a variety of resources and civil rights in their host city [13]. Guided by our literature review, we define internal migration status by comparing the current type (Shanghai versus not Shanghai) of household registration (‘hukou’). We analyzed mothers’ internal migrant status (Hukou) as a binary variable (1 = Shanghai-born women, 0 = Internal migrants). Sixty-three percent of the sample fell into the Shanghai native-born category (*n* = 83,872) and thirty-seven percent were internal migrant women (*n* = 49,486) out of the total sample of 133,358.

### Covariate variables and propensity score control variables

In all analyses, we adjusted for demographic and maternal clinical characteristics. Demographic covariates and relevant health variables were also controlled for in order to minimize potential selection bias associated with them, including maternal age at the child’s birth, age squared, gravida—the number of times a woman has been pregnant, birth parity—the number of pregnancies > 20 weeks, ethnicity (1 = Han Chinese; 0 = Ethnic Minority), nationality (1 = China; 0 = others), marriage status (1 = married; 0 = others), severe pregnant, i.e. whether the woman was a critically ill obstetric patient (1 = obstetric critically ill; 0 = not critically ill), cesarean delivery (1 = cesarean delivery; 0 = natural delivery), occupation (1 = employed; 0 = not employed) and insurance status (1 = have health insurance; 0 = without any insurance).

### Analytical methods

Our analysis consists of two parts. We start with a series of logistic regression models, then, the propensity score matching (PSM) method was used to predict the competing likelihood of having a high-risk condition in pregnancy.

To address potential selection bias, previous studies have used several methods, including instrument variable (IV) analysis and the propensity score matching (PSM) approach [32]. Because it was not easy to find an ideal instrument for migration status, and because the PSM approach works well when dealing with a large data set, such as that used in our study with 133,358 live births over the study period, we selected the PSM approach. The propensity score matching (PSM) approach has been used in several fields to address issues of selectivity bias, heterogeneity and endogeneity [37], and it has been shown to offer estimates that address each potential source of confounding [38–40].

In our study, the outcome variable was the likelihood of a high-risk pregnancy with its determinants estimated through the use of logistic regression methods after controlling for confounding through the use of propensity score methods. The propensity score method was based on a two-stage procedure. In the first stage, the propensity score was estimated in order to identify those characteristics in the dataset that were associated with being a migrant woman. This estimated propensity score was used to match observations in the whole dataset in such a way that individuals with similar values to the identified determining covariates were grouped together in order to assess the independent effect of internal migrant status (Hukou), thereby controlling for potential confounding. In the second stage, the effect of internal migrant status (Hukou) on high-risk pregnancies was estimated using matching techniques based on propensity score estimation. Specifically, we implement each of three different matching methods to assess how sensitive our estimates were to each method: one-on-one matching; caliper matching; and nearest neighbour matching. The parameter we estimated was the average treatment effect (ATE) [41, 42], which in our study context refers to the independent effect of migrant status (Hukou) on the likelihood of a high-risk pregnancy. All estimates were performed using STATA 14.

## Results

### Data description

Table 1 provides descriptive statistics for each of the variables used in the study. Internal migrant status (Hukou), occupation, marriage status, insurance status, nationality, ethnicity, high-risk pregnancy, severe pregnant, and cesarean delivery were coded as binary variables. For example, if the mother was employed, married, had health insurance, not Shanghai-born Han Chinese women, who were high risk, severely pregnant and had a cesarean delivery, then each of these nine binary variables were all one.

**Table 1:** Summary statistics on the characteristics of the study population (Shanghai-native vs. Internal migrants)

Variable	All (*N* = 133,358)		Shanghai-native (*N* = 83,872)		Internal migrants (*N* = 49,486)	
	Mean	S.D	Mean	S.D	Mean	S.D
High-risk pregnancy	0.537	0.499	0.523	0.499	0.561	0.496
Age	30.69	3.911	31.01	3.864	30.16	3.933
Age square	957.4	247.2	976.5	247.2	925.2	243.7
Ethnic group	0.986	0.116	0.992	0.0911	0.978	0.148
Nationality	0.998	0.0488	0.999	0.036	0.996	0.0649
Occupation	0.938	0.241	0.964	0.186	0.894	0.308
Marriage status	0.998	0.0439	0.999	0.036	0.997	0.0548
Insurance status	0.615	0.487	0.678	0.467	0.508	0.5
Gravida	1.81	1.079	1.725	1.006	1.955	1.179
Parity	1.25	0.455	1.225	0.427	1.293	0.497
Severe pregnant	0.00112	0.0334	0.000942	0.0307	0.00141	0.0376
Cesarean delivery	0.407	0.491	0.425	0.494	0.377	0.485

The average age of the sample of mothers was 30.7 years, with 37% being internal migrants. Just over half (54%) of the mothers had a high-risk pregnancy. Almost all (94%) of the mothers were employed, 99% were married and 62% had public insurance. In terms of nationality and ethnicity, almost all (99%) mothers were Han Chinese. And almost half (41%) of the women had a cesarean delivery.

### Logistic regression estimates

Logistic regression was used to estimate the relationship between internal migrant status (Hukou) and the likelihood of having a high-risk pregnancy after controlling for pregnancy status and socio-economic factors, but without imposing propensity score matching. The results are shown in Table 2. The marginal effects demonstrate that there is a high and statistically significant positive effect of internal migrant status (Hukou) on the likelihood of a high-risk pregnancy over the entire study period. We found that infants born to internal migrant women were more likely to be classified as a high-risk pregnancy by 0.038 (column 1, *p* < 0.001) than their urban counterparts. After controlling for all key explanatory variables, the effect of internal migrant status (Hukou) on the likelihood of a high-risk pregnancy remained significant at 0.050 to 0.060 (columns 3 and 5, *p* < 0.001). Notably, several covariates were strongly associated with the dependent variable (Table 2). A prior pregnancy, maternal gravitas, severe pregnant had a strong positive effect on the likelihood of having a high-risk pregnancy. Similarly, cesarean delivery was more likely to be associated with a high-risk pregnancy than natural vaginal birth. Besides, medical insurance and ethnicity had a strong negative effect on having a high-risk pregnancy.

**Table 2:** Logistic regression of the effect of internal migrant status (Hukou) on the rate of high-risk pregnancies

Variable	Model 1		Model 2		Model 3	
	Marginal effects	*P*-value	Marginal effects	*P*-value	Marginal effects	*P*-value
	(1)	(2)	(3)	(4)	(5)	(6)
Migrant status	0.038***	< 0.001	0.050***	< 0.001	0.060***	< 0.001
Age			–0.019***	< 0.001	–0.013***	–0.004
Age square			0.001***	< 0.001	0.001***	< 0.001
Ethnic group			–0.061***	< 0.001	–0.067***	< 0.001
Nationality			0.070**	–0.026	0.059*	–0.07
Occupation			–0.017***	–0.004	0.003	–0.676
Marriage status			0.005	–0.871	0.018	–0.579
Insurance status			–0.059***	< 0.001	–0.046***	< 0.001
Gravida					0.015***	< 0.001
Parity					0.004	–0.32
Severe pregnant					0.258***	< 0.001
Cesarean delivery					0.262***	< 0.001
Observations (N)	133,358	133,358	133,358	133,358	133,358	133,358

^*^ *p* < 0.05, ^**^ *p* < 0.01, ^***^ *p* < 0.001

### Propensity score matching estimates

To reduce the potential for selection bias, we used propensity score matching (PSM) methods to compare the women who are born in Shanghai to those that moved from other parts of China to Shanghai. Table 3 reports the average treatment effect (ATE) in terms of internal migrant status (Hukou) resulting from three different matching methods, all of which address the issue of confounding. The three methods are (1) one-on-one matching (column 1, row 1); (2) caliper matching (column 1, row 2); and (3) near neighbour matching (column 1, row 3). The estimates of the average treatment effect (ATE) demonstrate that internal migrant status (Hukou) increased the likelihood of high-risk pregnancies by 0.067 (column 1, row 1–3, *P* < 0.001). The propensity score matching methods yield findings that are almost consistent with the logistic regression results, after controlling for all key explanatory variables, shown in Table 2.

**Table 3:** Propensity score matching methods of the effect of internal migrant status (Hukou) on the rate of high-risk pregnancies

Outcome	ATE	*P*-value
	(1)	(2)
One to one	0.067***	< 0.001
Caliper	0.067***	< 0.001
Nearest Neighbour	0.067***	< 0.001
Observations (N)	111,108	111,108

This is an abridged set of results, and ATE is for internal migrant status (Hukou)

^*^ *p* < 0.05, ^**^ *p* < 0.01, ^***^ *p* < 0.001

The current literature suggests that migrant mothers tend to be isolated from the rest of society and are less likely to access maternal care which in turn results in their higher likelihood of a high-risk pregnancy, especially for their first child [43, 44]. Besides, under China’s Hukou system, internal migrant mothers are denied urban insurance and often have inferior employment prospects than local residents [10, 11]. Based on these factors, we extended our study by stratifying by birth parity, maternal employment status, and maternal insurance status. We did not stratify by maternal age and marital status since maternal age above 35 years of age was one of the criteria used to define a high-risk pregnancy, and the overwhelming majority of our sample were married (99%).

### Sub-sample analysis using propensity score matching estimates

When we divided the sample into sub-samples by birth parity, maternal employment status, and maternal insurance status, the results were robust. Table 4 reports the results of the effect of internal migrant status (Hukou) on the likelihood of a high-risk pregnancy stratified by birth parity, employment status, and insurance status, using propensity score matching estimates.

**Table 4:** The effect of the internal migrant status (Hukou) on the rate of high-risk pregnancies stratified by birth parity, occupation, and insurance, propensity score matching methods

Outcome	ATE	*P*-value	Observations (N)
	(1)	(2)	(3)
Panel A: stratified by birth parity			
First birth	0.066***	< 0.001	101,236
2nd or higher birth	0.027***	< 0.001	32,122
Panel B: stratified by employment status			
Employed	0.058***	< 0.001	125,076
Not employed	0.037***	< 0.001	8,282
Panel C: stratified by insurance status			
Have insurance	0.094***	< 0.001	82,038
Without any insurance	-0.005	0.256	51,320

The table shows the effect of internal migrant status on high-risk pregnancy. Each panel presents the results of the propensity score matching approach stratified by birth parity (Panel A), employment status (Panel B), and insurance status (Panel C)

^*^ *p* < 0.05, ^**^ *p* < 0.01, ^***^ *p* < 0.001

We first report the results stratified by birth parity shown in Panel A of Table 4; the results show that internal migrant mothers who give birth for the first time have a higher likelihood by 0.066 (column 1, row 1, *P* < 0.001) of having high-risk pregnancies than non-migrants having their first child. While internal migrant mothers having their second (or higher order of birth) still had a higher likelihood of having high-risk pregnancies than non-migrants, their higher likelihood was smaller than that for a mother having their first child, 0.027 (column 1, row 2, *P* < 0.001) compared to 0.066 (column 1, row 1). A possible reason for these results is a lack of access to prenatal health education among internal migrant mothers that may expose them to high-risk pregnancies. However, internal migrant mothers who are having their second (or higher) birth may be sufficiently experienced to make up this deficiency in their lack of access to prenatal care and education thereby lowering their risk of having a high-risk pregnancy relative to those having their first child.

The results of the analysis stratified by employment status are shown in Panel B of Table 4, which demonstrate employed migrant mothers have a greater risk of having high-risk pregnancies by 0.058 (column 1, row 3, *P* < 0.001) than non-migrants who were employed. The difference persisted but was slightly smaller for those internal migrants that were not employed, who have the risk of having high-risk pregnancies by 0.037 (column 1, row 4, *P* < 0.001). Internal migrant women usually have poor employment circumstances and low economic status, which together result in worse health outcomes [44]. As such, employed internal migrants tend to represent women in low-paid and inferior-status positions that represent a risk factor for a high-risk pregnancy.

We also extended our results by insurance status. Panel C of Table 4 reports the results stratified by insurance status. Despite having insurance, internal migrants still have a greater likelihood of having a high-risk pregnancy by 0.094 (column 1, row 5, *P* < 0.001). In the case of those without insurance, there was no statistical difference between migrants and Shanghai-born mothers (column 1, row 6, *P* = 0.256). Most internal migrants were covered by insurance from their hometown, while China’s system of social health insurance and the Hukou system limited their opportunity to transfer their insurance across regions [45]. Some of them may hold a decent job in their host city and then obtain local insurance. However, insurance coverage still lags behind that for local residents [46]. Internal migrants consequently have a greater risk of having a high-risk pregnancy despite having insurance.

## Sensitivity tests

We examined how sensitive our results were to alternative estimation techniques and the manner in which some variables were defined. First, as mentioned previously, we used three alternative matching methods: one-on-one matching; caliper matching; and nearest-neighbor matching approaches. The results of all three matching methods were the same, as shown in Table 3. Moreover, as demonstrated in Additional file 1: Figure S1, there was sufficient overlap in the characteristics of the internal migrants and locally born residents to find adequate matches. Second, we examined whether our results were maintained when we stratified for birth parity, employment status, and insurance status. Almost all of these variables were statistically significant. Almost all of our findings were highly robust, as shown in Table 4 (*P* > 0.001).

## Discussion

### Key findings and implications

This study assessed the relationship between internal migrant status (Hukou) and the likelihood of having a high-risk pregnancy in the context of China’s Hukou system, using propensity score matching method to reduce for potential self-selection bias into the migration. The present study offers three key findings.

**First,** we identified a smaller, yet still significant, positive effect of internal migrant status (Hukou) on the likelihood of having a high-risk pregnancy. This result was maintained when we controlled for potential selection bias of migration. Specifically, we found that infants born to internal migrant women were more likely to be classified as a high-risk pregnancy by 0.067. Three alternative methods for controlling for potential selection bias resulted in similar results. This finding is consistent with prior studies in other countries [5, 9, 27, 28, 47, 48]. As mentioned earlier, China’s unique Hukou system denies access to public health services for internal migrant women, which may, in turn, be associated with their higher likelihood of a high-risk pregnancy.

**Second,** the association between internal migrant status (Hukou) and the occurrence of a high-risk pregnancy differed by maternal birth parity. Internal migrant mothers who were giving birth for the first time had a higher likelihood of having high-risk pregnancies compared to those internal migrant mothers who already had a prior delivery. This finding may be attributed to the fact that mothers who were having a second or subsequent child may have more experience than other mothers, thereby reducing their exposure to a high-risk pregnancy [43, 44].

**Third,** the difference in high-risk pregnancy persisted by maternal employment status, and maternal insurance status. When we stratified by employment status, those internal migrants who were employed were more likely to have high-risk pregnancies than those who were not employed. This observation may reflect the low pay and inferior employment status afforded to internal migrant mothers who were employed [49]. After stratifying by insurance coverage, internal migrants, even those with insurance, were still having a high-risk pregnancy possibly because the Hukou system may limit their opportunities to transfer their insurance across regions [45].

Our study represents a re-test of the “healthy migrant effect” for pregnant women in China. After addressing potential selection bias, we find that internal migrant women have a greater likelihood of having a high-risk pregnancy, which contrasts with the general observation that migrants report better health status than local residents. Our findings, however, are consistent with existing studies from Shanghai that report worse pregnancy conditions among internal migrants than those reported among their native-born counterparts [11]. This result may be due to the following reasons:

**First,** China’s Hukou system limits access to public health services, including urban health insurance for internal migrant workers in the host city. As a consequence, internal migrants face significant out-of-pocket costs to their use of such services, limiting their opportunity to receive maternal care that may, in turn, result in adverse reproductive outcomes with high-risk pregnancies being one such outcome [11, 22, 50].

**Second,** internal migrants, especially women, generally have low status, limited skills, and low-paying jobs, combined with an inability to afford access to medical care, which may further exacerbate disparities in health outcomes [44, 51]. Moreover, the lack of prenatal care awareness may result in an increased likelihood of a high-risk pregnancy [20, 50].

**Third,** studies also have suggested that internal migrants have a high likelihood of multiple abortions [52], as they are often younger and more likely to have premarital sex. Premarital childbearing is not common in China, therefore these young internal migrant girls are more likely to choose abortion if they were to become pregnant. These factors, when taken together, are important risk factors for a high-risk pregnancy [52].

To the best of our knowledge, this is the first study to use propensity score matching (PSM) methods to address the consequences of migration on the likelihood of having a high-risk pregnancy. Moreover, there very few studies that have focused on adverse maternal health outcomes associated with internal migration, particularly under China’s Hukou system.

### Strengths and limitations

In this study, we examine the relationship between internal migrant status (Hukou) and the likelihood of having a high-risk pregnancy in the context of China’s Hukou system. Our findings add to the growing body of evidence that internal migrant mothers in China are more likely to have high-risk pregnancies. First, the study used extensive, detailed, and high-quality medical records data from the Shanghai First Maternity and Infant Hospital. Second, our empirical estimates were based on a clear conceptual framework and addressed the potential for selection bias by using propensity score matching methods. Third, previous studies in China used national-level data, unlike our study that used patient-level health information. Our study was based on inpatient hospital records data, which includes comprehensive maternal medical diagnosis information. The findings remain robust when controlled for a wide range of socio-economic, demographic, and health-related covariates.

The current study has limitations. First, the hospital that was the centre for this study was based in Shanghai, and this hospital may not be representative of all hospitals in China. Consequently, our study findings may lack generalizability. Second, we were unable to directly test various other mechanisms (e.g., maternal psychological attributes, health lifestyle, and life satisfaction) that may account for some of the observed results as such data were not available. Third, women more than 35 years were included in the construct of a high-risk pregnancy. Age itself, was not an indicator of poor health, rather a demographic variable. However, mothers more than 35 years were more likely to have adverse birth outcomes so age does partially represent risk exposure to an adverse outcome [53, 54]. Fourth, we were not able to decompose the indicator for high-risk pregnancies indicator into separate factors. The interpretation of the results is not straightforward. However, high-risk pregnancies refers to a wide variety of medical and/or obstetric complications that are internationally-accepted as high-risk conditions during the course of pregnancy. These conditions are the main causes of maternal and infant mortality. Fifth, the inclusion of only women with live births may lead to bias in the rate of high-risk pregnancies as mothers who lost their babies during the course of pregnancy would not be included. However, because the Chinese government attached great importance to maternal and infant health over the whole course of pregnancy to lower the maternal and infant death rate. This increase in attention has been associated with a decline in infant mortality, such that the neonatal mortality rate (per 1,000 live births) in China reached 3.9 in 2019 [55], and in Shanghai, it reached 2.66 per 1,000 births in 2020 [56]. There is almost no death birth in this study period, therefore the statistic problem of infant death caused by the high-risk pregnancies may not be an issue. Sixth, the internal migrants in our study include internal migrants from other cities, such as Beijing, although they represent a very small subset (0.22%) of the data. Further research is needed to garner a more complete picture of maternal health and its potential interaction with the occurrence of high-risk pregnancies.

## Conclusion

This paper is the first study to describe the relationship between migration within China and inequities in the likelihood of high-risk pregnancies that may be attributed to China’s unique household registration (Hukou) system. The results suggest that even after accounting for a range of potential confounding variables through the use of the propensity score matching method and stratified by birth parity, employment status, and insurance coverage, internal migrant women still have an elevated likelihood of high-risk pregnancies than their urban counterparts in Shanghai.

These results contrast with the ‘healthy migrant hypothesis’ and suggest that internal migrants do not have health advantages in pregnancy outcomes as China’s unique Hukou system limits their access to public health services. This barrier to access public health services may be the key contributing factor associated with the elevated likelihood of high-risk pregnancies among internal migrant women.

## Supplementary Information

**Additional file 1: Figure S1.** Propensity score histograms by treatment status (untreated = Shanghai-native, treated = internal migrants).

## Data Availability

Our data will not be shared to protect the participants’ anonymity.

## Abbreviations

IV: Instrument variable
PSM: Propensity score matching
ATE: Average treatment effect
