# Supplementary material for: The relationship between internal migration and the likelihood of high-risk pregnancy: Hukou system and high-risk pregnancies in China
Source: BMC Pregnancy Childbirth. 2021 Jul 15;21:509. doi: 10.1186/s12884-021-03958-4 (PMC8283949; doi:10.1186/s12884-021-03958-4)
Supplement: Supplementary file 1 — Additional file 1: Figure S1. Propensity score histograms by treatment status (untreated = Shanghai-native, treated = internal migrants). [file 12884_2021_3958_MOESM1_ESM.docx]

**Additional file 1: Figure S1.** Propensity score histograms by treatment status (untreated = Shanghai-native, treated = internal migrants)

**
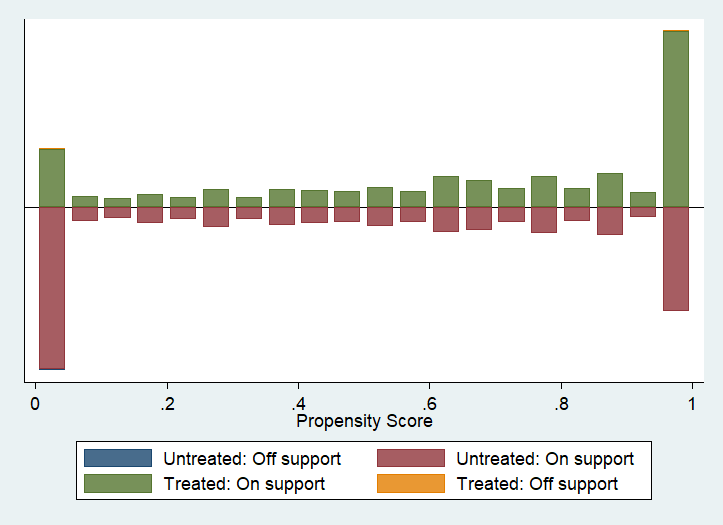
**

Note: The vertical axis marked in green refers to the treated group, while the untreated group is shown in red. Most of the observations are on support. Hence there is little loss of samples.
